# Six-Port Robotic Liver Resection Using Double Bipolar Clamp-Crush Method With Saline Drops: Advancing Safety, Efficiency, and Versatility in Liver Parenchymal Dissection

**DOI:** 10.7759/cureus.71580

**Published:** 2024-10-16

**Authors:** Noriyuki Egawa, Atsushi Miyoshi, Hiroki Koga, Kenji Kitahara, Hirokazu Noshiro

**Affiliations:** 1 Department of Surgery, Saga Medical Center Koseikan, Saga, JPN; 2 Department of Surgery, Saga University Faculty of Medicine, Saga, JPN

**Keywords:** clamp-crush technique, double bipolar method, liver parenchymal dissection, minimally invasive liver resection, robotic liver resection

## Abstract

Robotic liver resection (RLR) faces challenges in parenchymal dissection due to device limitations, necessitating the development of a safe, efficient, and versatile method for its widespread use.

We introduce our six-port RLR approach utilizing the double bipolar clamp-crush method with saline drops to overcome these device limitations. This method, combined with robotic bipolar forceps, maximizes the advantages of RLR by leveraging its multi-joint functionality and facilitates the dissection of strong, fibrotic liver tissue through the use of bipolar energy. The assistant surgeon strategically drops saline solution from an electrocautery system onto the operative field, while simultaneously removing crushed tissue, blood, and excess moisture using an endoscopic suction system through two assistant trocars. This process prevents tissue adhesion to the forceps and carbonization while maintaining a moist environment. Hemostasis is achieved using bipolar or monopolar coagulation under these conditions, with multiple hemostatic devices ensuring safety.

In our study, RLR was performed on 16 lesions in 13 patients, including two with cirrhosis and portal hypertension. Median blood loss was 27 mL, and no postoperative complications occurred. The forceps were changed a median of four times due to tissue adherence during liver parenchymal dissection, which prevented tissue adhesion and facilitated efficient hepatic resection.

Although further advances in instrumentation technology for RLR are needed, we are confident that our method will lower the barriers for many surgeons to adopt robotic liver resection and contribute to its further widespread use. Our six-port double bipolar clamp-crush method with saline drops shows promise for safe, efficient, and versatile liver parenchymal resection in current RLR practice.

## Introduction

Robotic surgery has become widely adopted in the field of gastrointestinal surgery and is now regarded as a standardized procedure. The technical advantages of robotic surgery - such as 3D vision, stable magnified images, EndoWrist instrumentation, physiological tremor filtering, and motion scaling - are fundamental in overcoming many limitations associated with laparoscopic surgery [1].

Robotic liver resection (RLR) refers to a surgical procedure in which robotic systems are employed to perform the resection of a portion of the liver. While RLR has gained significant popularity within liver surgery [2], its widespread adoption has been slow due to the delayed development of robotic devices suitable for liver parenchymal resection [3,4]. Specifically, devices such as the Cavitron Ultrasonic Surgical Aspirator (CUSA) and ultrasonic scalpels are commonly used for liver parenchymal transection in open and laparoscopic surgeries; however, robotic instruments that replicate these multi-jointed devices have yet to be developed.

In liver resection, performing liver parenchymal transection without causing bleeding is of utmost importance. Unfortunately, in RLR, surgeons cannot utilize the devices they are accustomed to. Therefore, to enhance the safety of RLR while capitalizing on its advantages, new approaches are essential for liver parenchymal transection.

Although several techniques have been reported [5-8], a standardized method for liver parenchymal dissection has yet to be established. To ensure the future safety and efficacy of RLR, it is crucial to overcome these device limitations and develop a safe, efficient, and versatile method for liver parenchymal dissection.

Two critical issues in liver parenchymal dissection must be addressed: preventing the adhesion of crushed liver tissue and carbonization to the forceps, and ensuring effective hemostasis. To tackle these challenges, we developed a six-port double bipolar clamp-crush technique with saline drops specifically for liver parenchymal dissection in RLR.

In this report, we provide a detailed description of our innovative technique and present its surgical outcomes.

## Technical report

Methods

In our liver parenchymal dissection technique in RLR, the console surgeon employs a double bipolar method to expand the surgical field, clamp and crush the liver parenchyma, dissect the vascular vessels, and achieve hemostasis. During the parenchymal dissection, the assistant surgeon uses two assist ports to drip saline solution onto the operative field through the tip attached to the ball-type electrode with a small tube for delivering saline solution, while simultaneously aspirating the crushed liver tissue and blood with an endoscopic aspiration system. This maintains an appropriately moist environment in the surgical field and removes crushed tissue and blood, thus preventing tissue and carbide from sticking to the robotic forceps and enabling efficient liver parenchymal dissection (Video 1).

**Video 1:** Usefulness of liver parenchymal resection with saline drops in robotic liver resection.

Furthermore, hemostasis can be achieved using the assistant’s laparoscopic device in addition to the robotic bipolar forceps, allowing multiple hemostatic devices to be used simultaneously and ensuring safe hemostatic operations.

Details of the surgical technique

**Figure 1:**
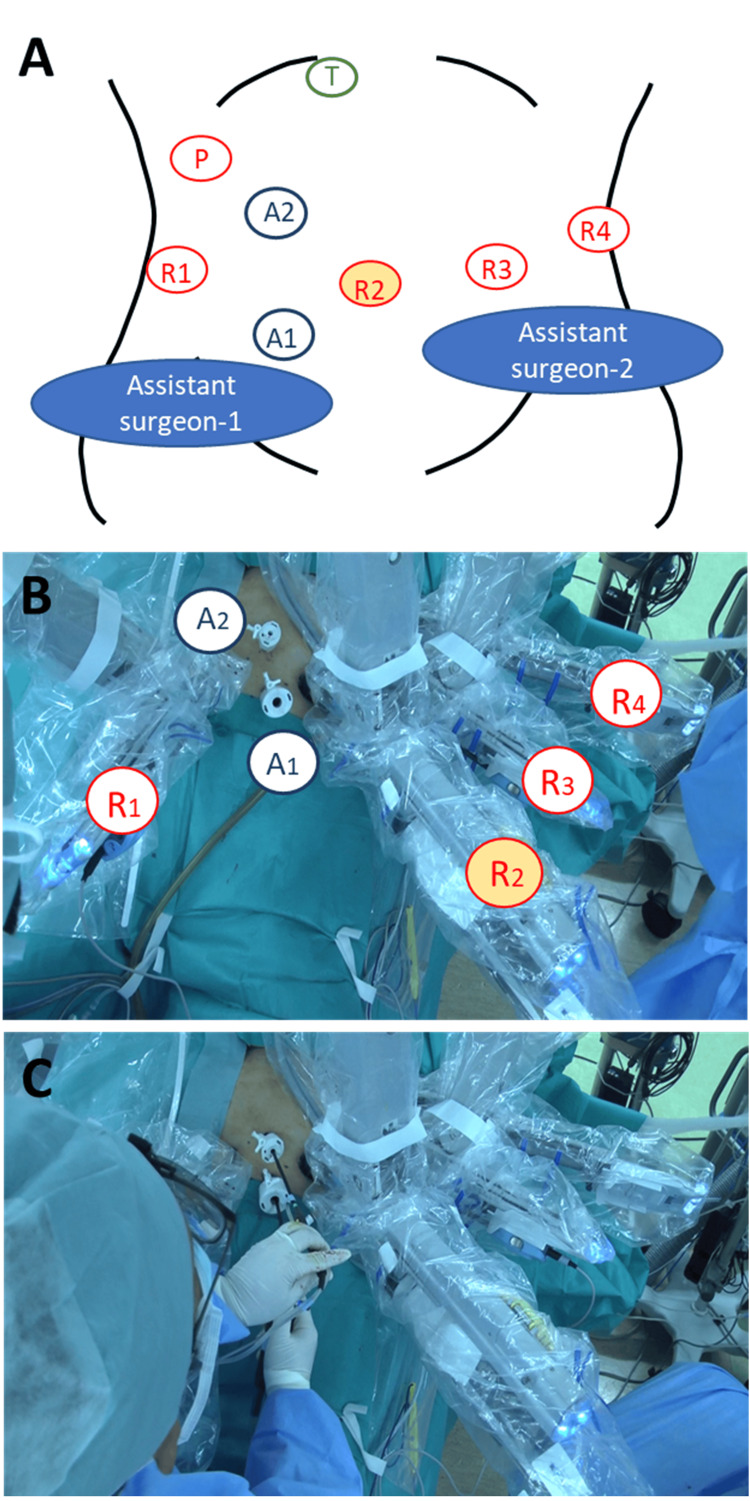
Trocar placement and intraoperative images showed the operating surgeon and the surgical robot for six-port robotic liver resection. Four robotic trocars were inserted concentrically around the target tumor. (A, B) A 12-mm trocar and a 5-mm trocar for the assistant surgeon were inserted between the No. 1 and 2 robotic trocars. (C) An assistant surgeon stood on the patient’s right side and inserted two endoscopic devices simultaneously through the two assist trocars. T: target tumor location, R1–4: robotic instrument arms, P: Pringle maneuver, A1: 12-mm trocar for assistant surgeon, A2: 5-mm trocar for assistant surgeon.

The patient was placed in the lithotomy position for left lobe tumors and in the left semi-decubitus position for right lobe tumors. All RLRs were performed using the da Vinci Surgical System (Intuitive Surgical, Inc., Sunnyvale, CA, USA) in this study. Four robotic trocars were inserted concentrically around the target tumor, with a 12-mm trocar and a 5-mm trocar for the assistant surgeon positioned between the robotic trocar in the lower right posterior ribcage area (No. 1) and the camera trocar (No. 2). Proper port placement is crucial to avoid interference between the assistant's forceps and the fenestrated bipolar forceps from robotic arm 1 inside the abdominal cavity, as well as between the assistant's body and robotic arm 1 outside the patient's body. Strategic positioning of the ports is essential to ensure that the forceps do not align parallel to the target. Additionally, increasing the separation between robotic arms 1 and 2 minimizes interference between the robotic arm and the assistant's body. Intermittent vascular occlusion of the hepatoduodenal ligament (Pringle's maneuver) was selectively applied during liver transection. Pringle’s maneuver preparations were made in all cases, with placement in the right upper abdomen for all tumor locations, allowing the first assistant to perform the maneuver effectively. Because the catheter for Pringle’s maneuver passed through the cephalad dorsal side of the robotic forceps, there was no interference between the forceps and the catheter. The first assistant surgeon stood on the patient’s right side (Figure 1) (Video 2).

**Video 2:** Intraoperative movements of the assistant surgeon and port placement considerations.

The robotic trocars were assigned as follows: No. 1 accommodated the operator’s left hand equipped with fenestrated bipolar forceps, No. 2 accommodated the 30-degree endoscope, No. 3 accommodated the operator’s right hand equipped with Maryland bipolar forceps, and No. 4 was used for liver retraction with Cadiere forceps.

**Figure 2:**
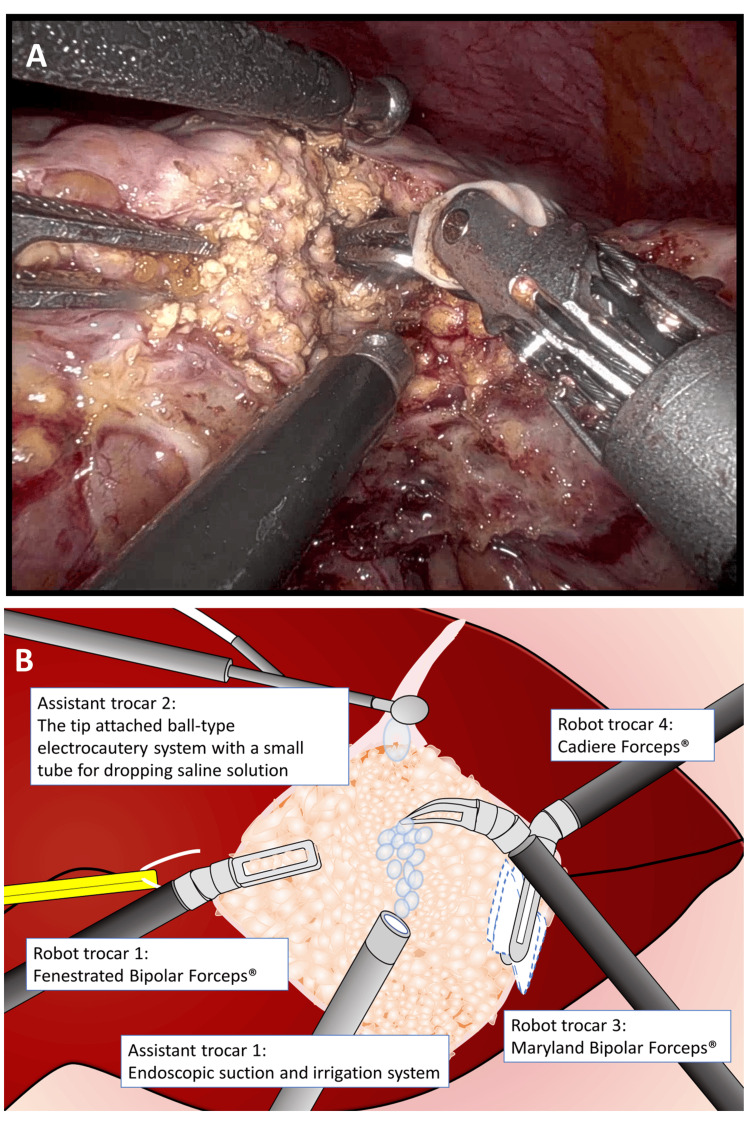
Intraoperative views of six-port robotic liver resection with double bipolar clamp-crush method with saline drops. Intraoperative photographs (A) and their schematics (B) of six-port robotic liver resection with double bipolar clamp-crush method with saline drops. Robotic trocar No. 1 accommodated the operator’s left hand equipped with fenestrated bipolar forceps, No. 2 accommodated the 30-degree endoscope, No. 3 accommodated the operator’s right hand equipped with Maryland bipolar forceps, and No. 4 was used for liver retraction using Cadiere forceps. The ball-tipped electrocautery system was inserted through a 5-mm trocar for the assistant, and an endoscopic suction system was inserted through a 12-mm trocar for the assistant. The Maryland bipolar forceps were used to dissect the liver parenchyma, fibrous tissue, and small vessels using the clamp-crush technique and the double bipolar method. The assistant surgeon continuously dripped saline solution onto the tip of the Maryland bipolar forceps from the tip of the ball-type electrocautery system and simultaneously removed these tissues, blood, and excess moisture using an endoscopic suction system.

The Maryland bipolar forceps were connected to an electrosurgical generator (VIO 3; Erbe Elektromedizin, Tübingen, Germany) with the ability to switch between bipolar auto-cut mode and bipolar soft-coagulation mode using an external foot pedal. The fenestrated bipolar forceps were plugged into an integrated electrosurgical unit (ERBE VIO dV; Erbe Elektromedizin) and used in bipolar soft-coagulation mode. A ball-tipped electrocautery system was set in soft-coagulation mode and inserted through a 5-mm trocar for the assistant, while an endoscopic suction system was set in a soft-coagulation mode and inserted through a 12-mm trocar for the assistant (Figure 2).

During liver parenchymal dissection, the Cadiere forceps from trocar No. 4 were used as a retraction arm. The fenestrated bipolar forceps maintained expansion of the surgical field, and the Maryland bipolar forceps were used to dissect the liver parenchyma with a clamp-crush maneuver using the double bipolar technique. In normal livers with minimal fibrosis, dissection could be easily achieved solely with the clamp-crush method by the Maryland bipolar forceps, without the need for bipolar energy. When a large vessel is identified, robotic clips are applied to ensure secure hemostasis. The multi-jointed functionality of the robotic forceps allows for precise clipping without obstructing the vessel’s direction. The assistant surgeon continuously dripped saline solution onto the tip of the Maryland bipolar forceps through the ball-type electrode to maintain an adequately moist surgical field and wash away crushed liver tissue and blood. Simultaneously, these materials were removed using the endoscopic suction system, preventing crushed tissue from adhering to the forceps. Additionally, removing crushed tissue in advance minimized carbonized tissue sticking to the forceps, reducing the need for forceps replacement and enabling efficient liver parenchymal dissection. Maintaining a moderately moist environment with saline drops also prevented excessive electrical resistance during hemostasis and carbonization of tissue, ensuring effective hemostasis (Video 3).

**Video 3:** Liver parenchymal resection in robotic liver resection for normal liver.

The advantage of using two assistant devices is that they allow for simultaneous saline irrigation and suction of tissue, blood, and excess fluids, enabling continuous saline dripping. If the flow of fluids relies solely on the laparoscopic suction-irrigation system, there will be no fluid supply while suctioning, which could result in tissue adhering to the forceps. However, a disadvantage is that the assistant must be somewhat familiar with laparoscopic surgery, as they need to coordinate with the robotic surgeon’s clamp-crush technique to effectively suction the tissue. Certainly, there are instances where the assistant is unable to use two devices simultaneously due to interference with the robotic arms. In such cases, the assistant must assess the situation and decide whether to prioritize saline irrigation or the suction of fragmented tissue and blood, and then proceed accordingly. The saline drip device rarely obstructs the surgical field of view during robotic liver resection. This is because saline can be administered from outside the field of vision, and it can be delivered along the surface of the liver or along the robotic forceps, allowing for fluid supply from multiple angles.

Fibrous tissue and small vessels were dissected in bipolar-cut mode after pre-coagulation in bipolar soft-coagulation mode, depending on tissue thickness, to facilitate bloodless dissection (Video 4).

**Video 4:** Liver parenchymal resection in robotic liver resection for the liver in chronic hepatitis.

In patients with severe parenchymal fibrosis due to cirrhosis, dissection using the clamp-crush method alone was challenging. However, in these cases, the use of bipolar energy to cut fibrous tissue enabled the resection of the severely fibrotic liver parenchyma.

For superficial bleeding, hemostasis could be achieved using bipolar energy in soft-coagulation mode with the robotic forceps. For deep bleeding that could not be controlled with bipolar forceps, hemostasis was immediately achieved in monopolar soft-coagulation mode with the ball-type electrocautery system or endoscopic suction system operated by the assistant surgeon. Four hemostatic devices were available to ensure immediate and safe hemostasis, with modes selected based on the site and nature of the bleeding (Video 5).

**Video 5:** Liver parenchymal resection in robotic liver resection for cirrhosis with portal hypertension.

Based on these principles, our six-port RLR technique enables liver parenchymal dissection across a wide range of cases, from normal liver to severe cirrhosis with advanced fibrosis, while ensuring safe and efficient hemostatic procedures during bleeding. This method also minimizes the need for forceps replacement due to scorching, thereby achieving efficient hepatic parenchymal dissection.

Results

Six-port RLR using the double bipolar clamp-crush method with saline drops was performed for 13 patients with 16 lesions at Saga Medical Center Koseikan in Japan from June 2022 to May 2024. Nine patients had hepatocellular carcinoma, and four had metastatic liver cancer. Two of the patients had liver cirrhosis. Patient characteristics and surgical outcomes are shown in Table 1.

**Table 1:** Patient characteristic and surgical outcomes

Case No.	Diagnosis＊	Procedure	Location	Liver status§	Operation time (min)	Console time (min)	Liver resection time (min)	Blood loss (g)	Pringle	Replacement forceps (times)	Conversion	Complication†	F-stage‡	Post-operative stay (day)
1	HCC	Partial	L (S3)	CH	182	96	64	13	-	2	-	-	3	7
2	MLC	Partial ×4	L,M (S2.S2/3,S3,S4)	NL	367	300	247	20	-	6	-	-	0	9
3	HCC	Partial	P (S6/7)	CH	165	108	37	27	-	1	-	-	3	10
4	HCC	Partial	L (S2)	CH	233	175	74	80	+	4	-	-	3	9
5	HCC	Partial	P (S6)	CH	314	180	81	74	-	5	-	-	2	9
6	MLC	Partial	L (S2)	NL	346	241	156	100	-	12	-	-	0	8
7	MLC	Partial	M (S4)	NL	298	151	101	34	+	2	-	-	0	9
8	HCC	Partial	A (S8)	CH	223	156	76	50	+	3	-	-	2	8
9	HCC	Partial	P (S6)	LC	329	240	162	410	+	9	-	-	4	9
10	HCC	Partial	A (S5)	LC	308	230	101	25	+	6	-	-	4	8
11	HCC	Partial	L (S3)	NL	251	195	57	20	-	3	-	-	0	9
12	HCC	Anatomical	L (S3)	CH	382	292	233	20	+	7	-	-	1	13
13	MLC	Partial	M (S4)	NL	243	178	84	15	+	3	-	-	0	14
Median					298	180	84	27		4				9
＊ HCC: hepatocellular carcinoma; MLC: metastatic liver cancer; § NL: normal liver; CH: chronic hepatitis; LC: liver cirrhosis † Clavien-Dindo Classification ≧ grade Ⅲ ‡ The degree of hepatic fibrosis was assessed by the New Inuyama classification														

The median console time was 180 minutes, and the median liver parenchymal dissection time was 84 minutes. The median blood loss was 27 mL, and no open or laparoscopic conversions were required. Furthermore, no perioperative complications occurred in any patient. Our procedure could be safely performed in patients with liver cirrhosis. Although the number of forceps changes was higher in patients with cirrhosis (Patients 9 and 10), the median number of robotic forceps changes due to tissue adherence during liver parenchymal dissection was four.

The surgical outcomes of robotic partial liver resection using other methods of liver parenchyma transection, as mentioned earlier, showed a median operative time of 211-485 minutes and blood loss of 5-256 mL [5-8]. Although a strict comparison is not possible due to differences in factors such as the background liver condition, number of resections, resection sites, and whether a simultaneous cholecystectomy was performed, our surgical outcomes were comparable to these results.

Our 6-port RLR was safely implemented in patients, including those with liver cirrhosis, demonstrating a wide range of applicability. Additionally, it enabled efficient liver parenchymal transection with minimal robotic forceps exchange.

## Discussion

Minimally invasive surgery (MIS), including laparoscopic and robotic procedures, has rapidly expanded in general surgery. Robotic surgery offers several advantages, such as tremor filtration, enhanced magnified views, and multi-jointed instruments. While the safety and efficacy of robotic surgery in general surgery are well-established, challenges remain regarding the cost-effectiveness of laparoscopic surgery, and further advancements in robotic surgery are expected [9,10]. However, in the context of liver resection, the widespread adoption of robotic liver surgery has been slow due to the delayed development of robotic devices suitable for liver parenchymal resection.

An international consensus statement on RLR was published in 2018 [11], and RLR has gradually gained popularity [2]. However, there are still few facilities that have introduced RLR, indicating the need for further research in this field [12]. Various methods of liver parenchymal transection have been reported for use in RLRs [5-8], however, an optimal method has not been established because of limitations of the devices used for liver parenchymal dissection [3,4]. Although liver parenchymal dissection by a Cavitron Ultrasonic Surgical Aspirator (CUSA) is widely used in open and laparoscopic liver surgery [13-15], CUSA as an instrument for robotic surgery has not been developed. Furthermore, laparoscopic liver parenchymal dissection using ultrasonic scalpels was performed widely [13,15], and the clamp-crush method of robotic liver parenchymal dissection using an ultrasonic scalpel has also been reported [5,6]. However, a disadvantage of liver parenchymal dissection using an ultrasonic scalpel is that it cannot perform the multi-joint function of the robotic forceps, and although it can handle linear dissection, it cannot adequately handle multidirectional dissection. In addition, the crushed tissue quickly sticks to the coated blade. A technique called “fusion surgery” has also been recently developed, in which an assistant surgeon performs liver transection using a CUSA [2,16] or ultrasonic scalpel or water jet [2,16,17]. Even with this method, however, the advantages of the articulated functionality of the robotic forceps are not fully demonstrated. These limitations highlight the need to develop a device that can handle liver parenchymal dissection in RLR.

The clamp-crush technique has been widely used in open liver resection and is familiar to most liver surgeons. Additionally, the multi-joint functionality of the robotic forceps is particularly advantageous and should be integral to RLR. Using Maryland bipolar forceps to dissect the liver parenchyma with the clamp-crush technique allows for maximal utilization of the robotic multi-joint function. There are no limitations in the joint range of motion, enabling dissection in any direction. We consider this feature to be the most valuable aspect of the clamp-crush technique in RLR. Moreover, in cases of severe fibrosis of the liver parenchyma, such as in patients with cirrhosis, the bipolar-cut mode proves more efficient for liver parenchymal dissection because the fibrotic tissue cannot be crushed with the clamp-crush technique alone. Therefore, we connected the Maryland bipolar forceps to an external generator, enabling the use of the bipolar-cut mode in conjunction with the clamp-crush method. This combination facilitates liver parenchymal dissection even in the presence of severe fibrosis, such as in liver cirrhosis. Additionally, the double bipolar method [18,19], characterized by the simultaneous use of Maryland bipolar forceps with the right hand and fenestrated bipolar forceps with the left hand, is highly advantageous in RLR. It allows for simultaneous liver parenchymal dissection, expansion of the surgical field, pre-coagulation of small vessels, and hemostasis during bleeding. Considering these factors, we have concluded that the double bipolar clamp-crush method is the most effective technique for liver parenchymal dissection in RLR. Therefore, we have chosen to implement this method in our introduction of RLR.

When crushed liver tissue adheres to the forceps during RLR, the forceps must be replaced. Because it takes time to replace the forceps, efficient parenchymal dissection cannot be achieved when tissue frequently adheres to the forceps. Therefore, we have used the saline drip method in RLR to prevent the crushed tissue itself and the carbide of the liver tissue and blood produced by the bipolar or monopolar energy from sticking to the forceps, as reported by Fujikawa and Kajiwara [7,8]. In their method, the robot was equipped with a suction instrument, and the console surgeon must perform continuous suctioning while simultaneously expanding the surgical field. Although highly effective, we found this technique to be particularly challenging in the introduction phase of robotic surgery.

Considering the aforementioned limitations, we developed a six-port RLR technique. In this approach, two assistant trocars are inserted to enable the simultaneous use of two laparoscopic devices by the assistant surgeon. This configuration enhances the concurrent application of saline dripping and aspiration, allowing the assistant surgeon to irrigate the surgical site with drops of saline while simultaneously aspirating and removing crushed liver tissue from the surgical field. In addition, maintaining an adequately moist environment prevents excessive electrical resistance due to bipolar or monopolar energy, thus minimizing tissue adherence to the forceps. Furthermore, the assistant performs the aspiration of free tissue, blood and excess moisture, allowing the console surgeon to concentrate on field expansion, liver resection, and hemostasis in the event of bleeding.

Hemostatic techniques are also important in liver resection procedures. The double bipolar method enables hemostasis using soft-coagulation mode from various angles with two robotic forceps, adapting to the nature and location of the bleeding point. However, managing deep bleeding can be challenging solely with bipolar soft-coagulation mode. In such cases, we employ the monopolar soft-coagulation mode with a ball-type electrocautery system or a laparoscopic suction system. Soft coagulation combined with saline application, as reported by Hirokawa et al. [20], has proven effective for achieving hemostasis in both open and laparoscopic hepatectomies. We consider it an indispensable system for RLR. Furthermore, in our six-port technique, saline is consistently dripped from the ball-type electrode with a small tube, ensuring the readiness of the monopolar soft-coagulation mode. We believe one of the advantages of six-port RLR is the ability to promptly initiate monopolar soft-coagulation in cases of deep bleeding challenging to address using robotic bipolar soft-coagulation alone. Four hemostatic devices (two robotic bipolar forceps, an assistant ball-type electrocautery system, and a suction system) can be deployed to manage bleeding from multiple angles according to the bleeding situation. Additionally, maintaining an adequately moist environment facilitates effective hemostasis.

A limitation of this study is the small sample size of 13 cases. The difficulty of liver resection varies greatly depending on the resection site, making it difficult to definitively conclude that our surgical procedure can be broadly applied to all RLRs. Moreover, if multi-jointed ultrasonic scalpels or CUSA instruments specifically designed for robotic surgery are developed, RLR techniques utilizing these tools could become the optimal approach.

## Conclusions

In conclusion, robotic liver resection (RLR) is gaining popularity, yet device limitations in liver parenchymal dissection underscore the need for new techniques. While further advancements in RLR instrumentation, such as a CUSA with multi-joint functionality, are necessary, we are confident that our approach will help lower the barriers for many surgeons to adopt robotic liver resection, thereby promoting its wider use.

Our study introduces a six-port RLR technique that utilizes a double bipolar clamp-crush method, combined with saline drops, to enable safe, efficient, and versatile liver parenchymal dissection. This method minimizes tissue adherence to the robotic forceps, reduces the frequency of forceps replacement, and ensures effective hemostasis, even in cases of severe liver cirrhosis. The technique has demonstrated broad applicability and safety across various clinical cases, suggesting its potential to become one of the standardized techniques in robotic liver resection.
